# Ethyl 1′′-benzyl-2′′-oxo-2′,3′,5′,6′,7′,7a’-hexa­hydro-1′*H*-di­spiro­[indeno­[1,2-*b*]quinoxaline-11,2′-pyrrolizine-3′,3′′-indoline]-1′-carboxyl­ate monohydrate

**DOI:** 10.1107/S1600536813011537

**Published:** 2013-05-11

**Authors:** Piskala Subburaman Kannan, Srinu Lanka, Sathiah Thennarasu, Gopal Vimala, Arunachalathevar SubbiahPandi

**Affiliations:** a Department of Physics, S.M.K. Fomra Institute of Technology, Thaiyur, Chennai 603 103, India; b Organic Chemistry Division, CSIR-Central Leather Research Institute, Adyar, Chennai 600 020, India; c Department of Physics, Presidency College (Autonomous), Chennai 600 005, India

## Abstract

In the title compound, C_38_H_32_N_4_O_3_·H_2_O, the quinoxaline–indene and pyrrolizine systems are essentially planar, with maximum deviations from their mean planes of 0.162 and 0.563 Å, respectively. The pyrrolizine ring forms dihedral angles of 88.53 (5) and 89.95 (8)° with the quinoxaline–indene system and the indoline ring, respectively. The central pyrrolidine ring has an envelope conformation with the C atom bearing the quinoxaline-indene system as the flap. The pyrrolidine ring of the indole system adopts an envelope conformation with the C atom bonded to the pyrrolizine ring N atom as the flap. The five-membered ring attached to the central pyrolidine ring adopts a twisted conformation. In the crystal, O—H⋯N and O—H⋯O hydrogen bonds between water mol­ecules and pyrrolizine N and carbonyl O atoms together with C—H⋯O inter­actions result in chains along [100].

## Related literature

For general background to spiro compounds and their biological activity, see: Pradhan *et al.* (2006 ▶); Saeedi *et al.* (2010 ▶); Dandia *et al.* (2011 ▶); He *et al.* (2003 ▶). For uses of pyrrolidine and quinoxaline derivatives, see: Amal Raj *et al.* (2003 ▶); Zarranz *et al.* (2003 ▶). For a related structure, see: Srinivasan *et al.* (2012 ▶). For ring conformations, see: Cremer & Pople (1975 ▶). For details of the synthesis, see: Azizian *et al.* (2005 ▶). 
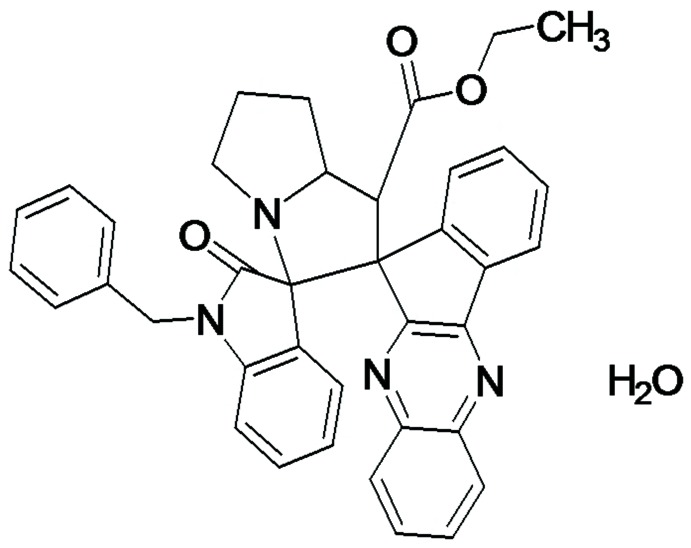


## Experimental

### 

#### Crystal data

C_38_H_32_N_4_O_3_·H_2_O
*M* *_r_* = 610.69
Triclinic, 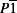

*a* = 11.0527 (3) Å
*b* = 11.5834 (3) Å
*c* = 12.2015 (3) Å
α = 97.370 (1)°
β = 92.037 (1)°
γ = 96.810 (1)°
*V* = 1536.25 (7) Å^3^
*Z* = 2
Mo *K*α radiation
μ = 0.09 mm^−1^
*T* = 293 K
0.25 × 0.22 × 0.19 mm

#### Data collection

Bruker APEXII CCD area detector diffractometer
Absorption correction: multi-scan (*SADABS*; Bruker, 2008 ▶) *T*_min_ = 0.979, *T*_max_ = 0.984
21165 measured reflections
5410 independent reflections
4751 reflections with *I* > 2σ(*I*)
*R*_int_ = 0.027

#### Refinement

*R*[*F* ^2^ > 2σ(*F* ^2^)] = 0.040
*wR*(*F* ^2^) = 0.114
*S* = 1.02
5410 reflections
422 parameters
3 restraints
H atoms treated by a mixture of independent and constrained refinement
Δρ_max_ = 0.27 e Å^−3^
Δρ_min_ = −0.31 e Å^−3^

### 

Data collection: *APEX2* (Bruker, 2008 ▶); cell refinement: *SAINT* (Bruker, 2008 ▶); data reduction: *SAINT*; program(s) used to solve structure: *SHELXS97* (Sheldrick, 2008 ▶); program(s) used to refine structure: *SHELXL97* (Sheldrick, 2008 ▶); molecular graphics: *ORTEP-3 for Windows* (Farrugia, 2012 ▶); software used to prepare material for publication: *SHELXL97* and *PLATON* (Spek, 2009 ▶).

## Supplementary Material

Crystal structure: contains datablock(s) global, I. DOI: 10.1107/S1600536813011537/bx2437sup1.cif

Structure factors: contains datablock(s) I. DOI: 10.1107/S1600536813011537/bx2437Isup2.hkl

Additional supplementary materials:  crystallographic information; 3D view; checkCIF report

## Figures and Tables

**Table 1:** Hydrogen-bond geometry (Å, °)

*D*—H⋯*A*	*D*—H	H⋯*A*	*D*⋯*A*	*D*—H⋯*A*
O4—H4*A*⋯N2	0.99 (2)	2.03 (2)	2.996 (2)	164 (2)
O4—H4*B*⋯O1^i^	0.99 (2)	2.31 (2)	3.222 (2)	154 (2)
C2—H2⋯O1^ii^	0.93	2.50	3.393 (2)	162
C24—H24*A*⋯O4^iii^	0.97	2.56	3.495 (3)	163

Symmetry codes: (i) 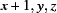; (ii) 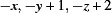; (iii) 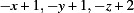.

**Fig. 1.**
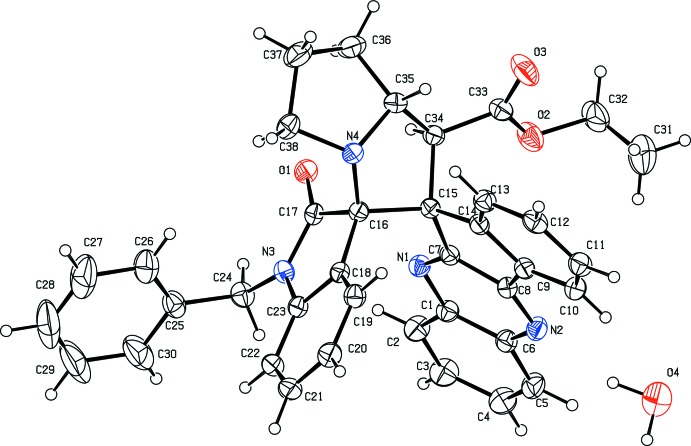
The molecular structure of the title compound with the atom numbering scheme. Displacement ellipsoids are drawn at the 30% probability level. H atoms are presented as small spheres of arbitrary radius.

**Fig. 2.**
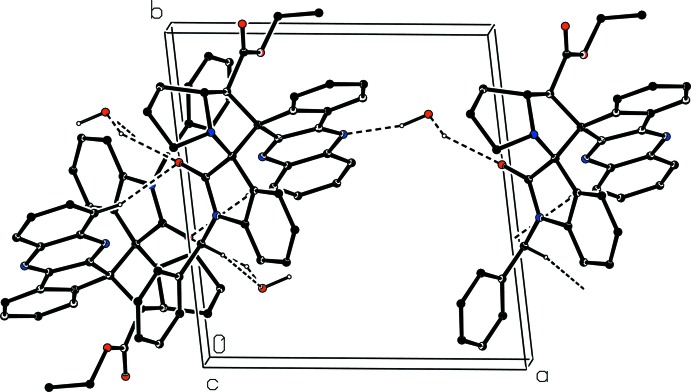
The molecular packing viewed down the c axis. Dashed lines shows the intermolecular O-H···O and C-H···O hydrogen bonds.

## supplementary crystallographic information

### Comment

Spiro compounds have received considerable interest due to their highly biological properties (Pradhan *et al.*, 2006); Thus more and more novel spiroheterocycle compounds have been prepared and characterized (Saeedi *et al.*, 2010); Dandia *et al.*, 2011). In addition , quinoxaline derivatives also showed various biological activites (He *et al.*, 2003).

Pyrrolidine derivatives are found to have anticonvulsant, antimicrobial and antifungal activities against various pathogens (Amal Raj *et al.*, 2003). Quinoxaline derivatives shown antibacterial, antiviral and anticancer properties (Zarranz *et al.*, 2003). As spiro pyrrolidine compounds are of great medicinal properties, we have undertaken the three dimensional structure of the title compound. In view of these importance and continuation of our work on the crystal structure analyis of pyrrolidine and quinoxaline derivatives, the crystal structure of the title compound has been carried out and the results are presented here.

X-Ray analysis confirms the molecular structure and atom connectivity of the compound as illustrated in Fig. 1. The quinoxaline-indene systems (C1-C15/N1-N2) and pyrrolizine system (C15-C16/C34-C38/N4), are essentially planar, with maximum deviations from mean plane of -0.162 Å for C3 atom and -0.563 Å for C36 atom, respectively.

The pyrrolizine ring (C15-C16/C34-C38/N4) forms a dihedral angles of 88.53 (5) and 89.95 (6)° with quinoxaline-indene systems and indole ring (C16-C23/N3) respectively. This clearly shows that the quinoxaline-indene ring system and indole rings are almost perpendicular to the pyrrolizine ring. The central pyrrolidine ring (C15-C16/C34-C35/N4) is enveloped on C15 with the puckering parameters of q_2_ = 0.4478 (2) Å, φ = 246.90 (2)° (Cremer & Pople, 1975). The pyrrolidine (C16-C18/C23/N3) of indole ring adopts envelope conformation on C16 with the puckering parameters of q_2_ = 0.0729 (2) Å, φ = 243.08 (1)°. The five membered ring (C35-C38/N4) attached with the central pyrolidine ring adopts twisted conformation on C36-C37 with the puckering parameters of q_2_ = 0.3767 (2) Å, φ = 92.96 (3)°.

In the crystal intra and intermolcular O-H···O hydrogen bonds between water molecules and pyrrolizine fragment N and carbonyl group O atoms of organic compound together with C-H···O interactions result in one-dimensional supramolecular structure.

### Experimental

Benzyl Isatin( 0.25 mmol), L-proline ( 0.3 mmol), Ethyl Indeno[1,2-b] quinoxalin-11-ylideneacetate( 0.25 mmol) (Azizian *et al.*, 2005) in ethanol refluxed for 60 min. The progress of the reaction was followed by TLC. After completion, the solvent was removed under reduced pressure and the resulting crude product was subjected to column chromatography. The product was recrystallised from methanol. Single crystals suitable for X-ray diffraction were obtained by slow evaporation of the solution of the title compound in methanol at room temperature.

### Refinement

All H atoms were fixed geometrically and allowed to ride on their parent C atoms, with C—H distances fixed in the range 0.93–0.97 Å with *U*_iso_(H) = 1.5*U*_eq_(C) for methyl H 1.2*U*_eq_(C) for other H atoms. The positions of methyl hydrogens were optimized rotationally.

### Crystal data

**Table:**

C_38_H_32_N_4_O_3_·H_2_O	*Z* = 2
*M**_r_* = 610.69	*F*(000) = 644
Triclinic, *P*1	*D*_x_ = 1.320 Mg m^−^^3^
Hall symbol: -P 1	Mo *K*α radiation, λ = 0.71073 Å
*a* = 11.0527 (3) Å	Cell parameters from 5410 reflections
*b* = 11.5834 (3) Å	θ = 1.7–25.0°
*c* = 12.2015 (3) Å	µ = 0.09 mm^−^^1^
α = 97.370 (1)°	*T* = 293 K
β = 92.037 (1)°	Block, colourless
γ = 96.810 (1)°	0.25 × 0.22 × 0.19 mm
*V* = 1536.25 (7) Å^3^	

### Data collection

**Table:**

Bruker APEXII CCD area detector diffractometer	5410 independent reflections
Radiation source: fine-focus sealed tube	4751 reflections with *I* > 2σ(*I*)
Graphite monochromator	*R*_int_ = 0.027
ω and φ scans	θ_max_ = 25.0°, θ_min_ = 1.7°
Absorption correction: multi-scan (*SADABS*; Bruker, 2008)	*h* = −13→13
*T*_min_ = 0.979, *T*_max_ = 0.984	*k* = −13→13
21165 measured reflections	*l* = −14→14

### Refinement

**Table:**

Refinement on *F*^2^	Secondary atom site location: difference Fourier map
Least-squares matrix: full	Hydrogen site location: inferred from neighbouring sites
*R*[*F*^2^ > 2σ(*F*^2^)] = 0.040	H atoms treated by a mixture of independent and constrained refinement
*wR*(*F*^2^) = 0.114	*w* = 1/[σ^2^(*F*_o_^2^) + (0.0592*P*)^2^ + 0.5303*P*] where *P* = (*F*_o_^2^ + 2*F*_c_^2^)/3
*S* = 1.02	(Δ/σ)_max_ = 0.001
5410 reflections	Δρ_max_ = 0.27 e Å^−^^3^
422 parameters	Δρ_min_ = −0.31 e Å^−^^3^
3 restraints	Extinction correction: *SHELXL97* (Sheldrick, 2008), Fc^*^=kFc[1+0.001xFc^2^λ^3^/sin(2θ)]^-1/4^
Primary atom site location: structure-invariant direct methods	Extinction coefficient: 0.0134 (15)

### Special details

**Table:**

Geometry. All esds (except the esd in the dihedral angle between two l.s. planes) are estimated using the full covariance matrix. The cell esds are taken into account individually in the estimation of esds in distances, angles and torsion angles; correlations between esds in cell parameters are only used when they are defined by crystal symmetry. An approximate (isotropic) treatment of cell esds is used for estimating esds involving l.s. planes.
Refinement. Refinement of F^2^ against ALL reflections. The weighted R-factor wR and goodness of fit S are based on F^2^, conventional R-factors R are based on F, with F set to zero for negative F^2^. The threshold expression of F^2^ > 2sigma(F^2^) is used only for calculating R-factors(gt) etc. and is not relevant to the choice of reflections for refinement. R-factors based on F^2^ are statistically about twice as large as those based on F, and R- factors based on ALL data will be even larger.

### Fractional atomic coordinates and isotropic or equivalent isotropic displacement parameters (Å^2^)

**Table:**

	*x*	*y*	*z*	*U*_iso_*/*U*_eq_	
C1	0.33289 (14)	0.60474 (13)	0.97720 (12)	0.0363 (3)	
C2	0.27983 (16)	0.53695 (15)	0.88023 (13)	0.0472 (4)	
H2	0.1967	0.5108	0.8754	0.057*	
C3	0.34944 (18)	0.50896 (17)	0.79262 (14)	0.0547 (5)	
H3	0.3132	0.4640	0.7286	0.066*	
C4	0.47454 (18)	0.54734 (16)	0.79835 (15)	0.0540 (5)	
H4	0.5207	0.5287	0.7378	0.065*	
C5	0.52933 (16)	0.61179 (15)	0.89196 (14)	0.0487 (4)	
H5	0.6127	0.6364	0.8951	0.058*	
C6	0.46058 (14)	0.64146 (13)	0.98405 (13)	0.0383 (3)	
C7	0.31818 (13)	0.69142 (12)	1.15216 (11)	0.0312 (3)	
C8	0.44754 (13)	0.72317 (12)	1.16075 (12)	0.0338 (3)	
C9	0.48255 (13)	0.77731 (12)	1.27325 (12)	0.0347 (3)	
C10	0.59802 (14)	0.81928 (14)	1.32171 (14)	0.0419 (4)	
H10	0.6670	0.8174	1.2803	0.050*	
C11	0.60769 (15)	0.86350 (14)	1.43215 (14)	0.0438 (4)	
H11	0.6839	0.8924	1.4660	0.053*	
C12	0.50493 (15)	0.86526 (13)	1.49326 (13)	0.0420 (4)	
H12	0.5134	0.8941	1.5682	0.050*	
C13	0.38954 (14)	0.82503 (13)	1.44535 (13)	0.0382 (3)	
H13	0.3210	0.8279	1.4873	0.046*	
C14	0.37783 (13)	0.78043 (12)	1.33380 (12)	0.0322 (3)	
C15	0.26261 (12)	0.73456 (12)	1.26003 (11)	0.0306 (3)	
C16	0.17216 (12)	0.64178 (12)	1.30948 (11)	0.0311 (3)	
C17	0.08006 (12)	0.57554 (12)	1.21443 (12)	0.0324 (3)	
C18	0.22870 (12)	0.54088 (12)	1.34875 (12)	0.0321 (3)	
C19	0.30618 (14)	0.53514 (14)	1.43864 (13)	0.0385 (3)	
H19	0.3328	0.6020	1.4881	0.046*	
C20	0.34386 (15)	0.42751 (15)	1.45399 (14)	0.0436 (4)	
H20	0.3963	0.4227	1.5141	0.052*	
C21	0.30422 (15)	0.32808 (14)	1.38100 (14)	0.0446 (4)	
H21	0.3322	0.2575	1.3915	0.054*	
C22	0.22365 (14)	0.33140 (13)	1.29237 (13)	0.0409 (4)	
H22	0.1954	0.2640	1.2441	0.049*	
C23	0.18692 (13)	0.43837 (12)	1.27831 (12)	0.0331 (3)	
C24	0.04034 (15)	0.37286 (14)	1.11225 (13)	0.0426 (4)	
H24A	0.1019	0.3390	1.0683	0.051*	
H24B	−0.0096	0.4104	1.0639	0.051*	
C25	−0.03895 (16)	0.27579 (15)	1.15617 (15)	0.0481 (4)	
C26	−0.10008 (19)	0.2954 (2)	1.25144 (18)	0.0668 (6)	
H26	−0.0929	0.3705	1.2906	0.080*	
C27	−0.1720 (3)	0.2049 (3)	1.2897 (3)	0.1052 (10)	
H27	−0.2130	0.2196	1.3544	0.126*	
C28	−0.1837 (3)	0.0943 (3)	1.2338 (4)	0.1271 (15)	
H28	−0.2319	0.0335	1.2602	0.153*	
C29	−0.1241 (4)	0.0736 (2)	1.1390 (4)	0.1244 (14)	
H29	−0.1321	−0.0018	1.1004	0.149*	
C30	−0.0512 (3)	0.16379 (19)	1.0990 (2)	0.0880 (8)	
H30	−0.0109	0.1487	1.0340	0.106*	
C31	0.4884 (3)	1.0676 (3)	1.1530 (2)	0.0950 (8)	
H31A	0.5302	1.1394	1.1343	0.143*	
H31B	0.5261	1.0025	1.1189	0.143*	
H31C	0.4930	1.0690	1.2319	0.143*	
C32	0.3595 (2)	1.05536 (18)	1.1130 (2)	0.0722 (6)	
H32A	0.3219	1.1220	1.1463	0.087*	
H32B	0.3549	1.0548	1.0334	0.087*	
C33	0.24386 (15)	0.95123 (13)	1.23835 (13)	0.0413 (4)	
C34	0.17726 (13)	0.83303 (12)	1.25340 (12)	0.0347 (3)	
H34	0.1176	0.8079	1.1911	0.042*	
C35	0.11039 (14)	0.83350 (13)	1.36057 (13)	0.0382 (3)	
H35	0.1548	0.8927	1.4170	0.046*	
C36	−0.02444 (16)	0.84985 (17)	1.35917 (17)	0.0558 (5)	
H36A	−0.0356	0.9317	1.3779	0.067*	
H36B	−0.0648	0.8201	1.2875	0.067*	
C37	−0.07122 (18)	0.77731 (19)	1.44790 (19)	0.0652 (5)	
H37A	−0.0531	0.8207	1.5212	0.078*	
H37B	−0.1586	0.7547	1.4375	0.078*	
C38	−0.00303 (15)	0.67035 (16)	1.43219 (15)	0.0481 (4)	
H38A	0.0062	0.6383	1.5012	0.058*	
H38B	−0.0456	0.6099	1.3772	0.058*	
N1	0.26028 (11)	0.63329 (11)	1.06378 (10)	0.0354 (3)	
N2	0.51881 (11)	0.70233 (11)	1.07875 (11)	0.0398 (3)	
N3	0.10044 (11)	0.46128 (10)	1.19862 (10)	0.0352 (3)	
N4	0.11669 (11)	0.71534 (11)	1.39436 (10)	0.0352 (3)	
O1	0.00192 (9)	0.61759 (9)	1.16524 (9)	0.0415 (3)	
O2	0.29325 (13)	0.94730 (11)	1.14075 (11)	0.0604 (4)	
O3	0.25075 (15)	1.03746 (11)	1.30440 (12)	0.0722 (4)	
O4	0.79032 (17)	0.76464 (16)	1.09100 (18)	0.0942 (6)	
H4A	0.7036 (15)	0.730 (2)	1.091 (2)	0.113*	
H4B	0.832 (2)	0.701 (2)	1.116 (2)	0.113*	

### Atomic displacement parameters (Å^2^)

**Table:**

	*U*^11^	*U*^22^	*U*^33^	*U*^12^	*U*^13^	*U*^23^
C1	0.0393 (8)	0.0371 (8)	0.0333 (8)	0.0066 (6)	0.0053 (6)	0.0052 (6)
C2	0.0447 (9)	0.0545 (10)	0.0399 (9)	0.0048 (7)	0.0013 (7)	−0.0019 (7)
C3	0.0626 (11)	0.0619 (11)	0.0365 (9)	0.0074 (9)	0.0049 (8)	−0.0054 (8)
C4	0.0621 (11)	0.0593 (11)	0.0406 (9)	0.0089 (9)	0.0191 (8)	0.0016 (8)
C5	0.0465 (9)	0.0528 (10)	0.0464 (10)	0.0018 (8)	0.0176 (8)	0.0050 (8)
C6	0.0406 (8)	0.0363 (8)	0.0384 (8)	0.0035 (6)	0.0085 (6)	0.0066 (6)
C7	0.0335 (7)	0.0279 (7)	0.0328 (7)	0.0038 (6)	0.0028 (6)	0.0059 (6)
C8	0.0332 (7)	0.0310 (7)	0.0374 (8)	0.0023 (6)	0.0045 (6)	0.0061 (6)
C9	0.0344 (8)	0.0301 (7)	0.0391 (8)	0.0021 (6)	0.0006 (6)	0.0046 (6)
C10	0.0332 (8)	0.0401 (8)	0.0507 (9)	0.0014 (6)	−0.0001 (7)	0.0037 (7)
C11	0.0389 (8)	0.0384 (8)	0.0514 (10)	0.0023 (7)	−0.0112 (7)	0.0015 (7)
C12	0.0499 (9)	0.0355 (8)	0.0387 (8)	0.0057 (7)	−0.0084 (7)	0.0004 (6)
C13	0.0410 (8)	0.0358 (8)	0.0371 (8)	0.0046 (6)	0.0024 (6)	0.0019 (6)
C14	0.0334 (7)	0.0265 (7)	0.0361 (8)	0.0021 (5)	−0.0002 (6)	0.0039 (6)
C15	0.0310 (7)	0.0286 (7)	0.0317 (7)	0.0028 (6)	0.0018 (6)	0.0026 (6)
C16	0.0294 (7)	0.0313 (7)	0.0322 (7)	0.0032 (6)	0.0021 (6)	0.0035 (6)
C17	0.0285 (7)	0.0349 (7)	0.0339 (7)	0.0003 (6)	0.0037 (6)	0.0074 (6)
C18	0.0290 (7)	0.0333 (7)	0.0346 (7)	0.0031 (6)	0.0051 (6)	0.0065 (6)
C19	0.0377 (8)	0.0400 (8)	0.0374 (8)	0.0020 (6)	0.0003 (6)	0.0065 (6)
C20	0.0402 (8)	0.0497 (9)	0.0443 (9)	0.0073 (7)	−0.0005 (7)	0.0174 (7)
C21	0.0446 (9)	0.0382 (8)	0.0552 (10)	0.0110 (7)	0.0062 (7)	0.0162 (7)
C22	0.0435 (9)	0.0325 (8)	0.0467 (9)	0.0045 (6)	0.0065 (7)	0.0044 (6)
C23	0.0326 (7)	0.0338 (7)	0.0335 (7)	0.0027 (6)	0.0059 (6)	0.0065 (6)
C24	0.0497 (9)	0.0412 (9)	0.0341 (8)	0.0005 (7)	0.0010 (7)	−0.0011 (7)
C25	0.0477 (9)	0.0398 (9)	0.0538 (10)	−0.0036 (7)	−0.0129 (8)	0.0074 (7)
C26	0.0652 (13)	0.0685 (13)	0.0669 (13)	−0.0072 (10)	0.0096 (10)	0.0227 (10)
C27	0.0797 (17)	0.122 (3)	0.120 (2)	−0.0198 (17)	0.0124 (16)	0.068 (2)
C28	0.089 (2)	0.103 (3)	0.190 (4)	−0.0445 (19)	−0.033 (2)	0.087 (3)
C29	0.126 (3)	0.0467 (14)	0.188 (4)	−0.0303 (16)	−0.045 (3)	0.0187 (19)
C30	0.1015 (19)	0.0468 (12)	0.1050 (19)	−0.0101 (12)	−0.0135 (15)	−0.0073 (12)
C31	0.0943 (19)	0.0915 (18)	0.100 (2)	−0.0146 (15)	0.0232 (15)	0.0334 (15)
C32	0.0932 (17)	0.0504 (11)	0.0784 (14)	0.0043 (11)	0.0299 (12)	0.0262 (10)
C33	0.0472 (9)	0.0336 (8)	0.0441 (9)	0.0077 (7)	0.0030 (7)	0.0066 (7)
C34	0.0356 (8)	0.0308 (7)	0.0378 (8)	0.0063 (6)	0.0006 (6)	0.0038 (6)
C35	0.0392 (8)	0.0346 (8)	0.0413 (8)	0.0092 (6)	0.0052 (6)	0.0022 (6)
C36	0.0458 (10)	0.0592 (11)	0.0681 (12)	0.0221 (8)	0.0132 (9)	0.0131 (9)
C37	0.0484 (11)	0.0759 (13)	0.0773 (14)	0.0196 (10)	0.0252 (10)	0.0170 (11)
C38	0.0409 (9)	0.0546 (10)	0.0496 (10)	0.0036 (7)	0.0150 (7)	0.0093 (8)
N1	0.0351 (6)	0.0369 (7)	0.0336 (7)	0.0039 (5)	0.0034 (5)	0.0030 (5)
N2	0.0353 (7)	0.0422 (7)	0.0405 (7)	−0.0001 (5)	0.0079 (6)	0.0035 (6)
N3	0.0369 (7)	0.0322 (6)	0.0347 (6)	−0.0002 (5)	−0.0005 (5)	0.0030 (5)
N4	0.0344 (6)	0.0350 (6)	0.0365 (7)	0.0054 (5)	0.0081 (5)	0.0039 (5)
O1	0.0350 (6)	0.0433 (6)	0.0458 (6)	0.0045 (5)	−0.0063 (5)	0.0076 (5)
O2	0.0844 (9)	0.0431 (7)	0.0546 (8)	0.0008 (6)	0.0227 (7)	0.0114 (6)
O3	0.1080 (12)	0.0355 (7)	0.0684 (9)	−0.0052 (7)	0.0259 (8)	−0.0036 (6)
O4	0.0750 (11)	0.0835 (12)	0.1217 (15)	−0.0009 (9)	0.0213 (11)	0.0098 (11)

### Geometric parameters (Å, º)

**Table:**

C1—N1	1.3795 (19)	C22—H22	0.9300
C1—C2	1.401 (2)	C23—N3	1.4106 (19)
C1—C6	1.421 (2)	C24—N3	1.4536 (19)
C2—C3	1.367 (2)	C24—C25	1.508 (2)
C2—H2	0.9300	C24—H24A	0.9700
C3—C4	1.398 (3)	C24—H24B	0.9700
C3—H3	0.9300	C25—C26	1.372 (3)
C4—C5	1.361 (3)	C25—C30	1.381 (3)
C4—H4	0.9300	C26—C27	1.380 (3)
C5—C6	1.408 (2)	C26—H26	0.9300
C5—H5	0.9300	C27—C28	1.362 (5)
C6—N2	1.370 (2)	C27—H27	0.9300
C7—N1	1.2994 (19)	C28—C29	1.361 (6)
C7—C8	1.430 (2)	C28—H28	0.9300
C7—C15	1.5234 (19)	C29—C30	1.392 (5)
C8—N2	1.3111 (19)	C29—H29	0.9300
C8—C9	1.453 (2)	C30—H30	0.9300
C9—C10	1.393 (2)	C31—C32	1.475 (4)
C9—C14	1.397 (2)	C31—H31A	0.9600
C10—C11	1.375 (2)	C31—H31B	0.9600
C10—H10	0.9300	C31—H31C	0.9600
C11—C12	1.381 (2)	C32—O2	1.458 (2)
C11—H11	0.9300	C32—H32A	0.9700
C12—C13	1.386 (2)	C32—H32B	0.9700
C12—H12	0.9300	C33—O3	1.193 (2)
C13—C14	1.388 (2)	C33—O2	1.325 (2)
C13—H13	0.9300	C33—C34	1.512 (2)
C14—C15	1.5329 (19)	C34—C35	1.524 (2)
C15—C34	1.5719 (19)	C34—H34	0.9800
C15—C16	1.5725 (19)	C35—N4	1.4879 (19)
C16—N4	1.4550 (18)	C35—C36	1.524 (2)
C16—C18	1.510 (2)	C35—H35	0.9800
C16—C17	1.5725 (19)	C36—C37	1.522 (3)
C17—O1	1.2171 (17)	C36—H36A	0.9700
C17—N3	1.3593 (19)	C36—H36B	0.9700
C18—C19	1.381 (2)	C37—C38	1.520 (3)
C18—C23	1.393 (2)	C37—H37A	0.9700
C19—C20	1.393 (2)	C37—H37B	0.9700
C19—H19	0.9300	C38—N4	1.4756 (19)
C20—C21	1.379 (2)	C38—H38A	0.9700
C20—H20	0.9300	C38—H38B	0.9700
C21—C22	1.384 (2)	O4—H4A	0.997 (17)
C21—H21	0.9300	O4—H4B	0.981 (16)
C22—C23	1.378 (2)		
			
N1—C1—C2	119.29 (14)	C25—C24—H24A	108.9
N1—C1—C6	121.71 (13)	N3—C24—H24B	108.9
C2—C1—C6	119.00 (14)	C25—C24—H24B	108.9
C3—C2—C1	120.44 (16)	H24A—C24—H24B	107.7
C3—C2—H2	119.8	C26—C25—C30	118.8 (2)
C1—C2—H2	119.8	C26—C25—C24	121.92 (16)
C2—C3—C4	120.58 (16)	C30—C25—C24	119.30 (19)
C2—C3—H3	119.7	C25—C26—C27	120.7 (3)
C4—C3—H3	119.7	C25—C26—H26	119.7
C5—C4—C3	120.48 (16)	C27—C26—H26	119.7
C5—C4—H4	119.8	C28—C27—C26	120.6 (3)
C3—C4—H4	119.8	C28—C27—H27	119.7
C4—C5—C6	120.45 (16)	C26—C27—H27	119.7
C4—C5—H5	119.8	C29—C28—C27	119.3 (3)
C6—C5—H5	119.8	C29—C28—H28	120.3
N2—C6—C5	119.23 (14)	C27—C28—H28	120.3
N2—C6—C1	121.73 (13)	C28—C29—C30	120.9 (3)
C5—C6—C1	119.02 (15)	C28—C29—H29	119.6
N1—C7—C8	123.16 (13)	C30—C29—H29	119.6
N1—C7—C15	126.77 (12)	C25—C30—C29	119.7 (3)
C8—C7—C15	110.07 (12)	C25—C30—H30	120.2
N2—C8—C7	123.60 (13)	C29—C30—H30	120.2
N2—C8—C9	127.85 (13)	C32—C31—H31A	109.5
C7—C8—C9	108.53 (12)	C32—C31—H31B	109.5
C10—C9—C14	121.61 (14)	H31A—C31—H31B	109.5
C10—C9—C8	129.58 (14)	C32—C31—H31C	109.5
C14—C9—C8	108.80 (12)	H31A—C31—H31C	109.5
C11—C10—C9	118.45 (15)	H31B—C31—H31C	109.5
C11—C10—H10	120.8	O2—C32—C31	110.82 (19)
C9—C10—H10	120.8	O2—C32—H32A	109.5
C10—C11—C12	120.43 (15)	C31—C32—H32A	109.5
C10—C11—H11	119.8	O2—C32—H32B	109.5
C12—C11—H11	119.8	C31—C32—H32B	109.5
C11—C12—C13	121.47 (15)	H32A—C32—H32B	108.1
C11—C12—H12	119.3	O3—C33—O2	123.94 (15)
C13—C12—H12	119.3	O3—C33—C34	125.11 (15)
C12—C13—C14	118.96 (15)	O2—C33—C34	110.94 (13)
C12—C13—H13	120.5	C33—C34—C35	113.68 (12)
C14—C13—H13	120.5	C33—C34—C15	114.17 (12)
C13—C14—C9	119.07 (13)	C35—C34—C15	103.24 (11)
C13—C14—C15	129.73 (13)	C33—C34—H34	108.5
C9—C14—C15	111.17 (12)	C35—C34—H34	108.5
C7—C15—C14	100.96 (11)	C15—C34—H34	108.5
C7—C15—C34	115.65 (11)	N4—C35—C36	105.46 (13)
C14—C15—C34	111.25 (11)	N4—C35—C34	105.26 (11)
C7—C15—C16	116.12 (11)	C36—C35—C34	119.23 (14)
C14—C15—C16	114.20 (11)	N4—C35—H35	108.8
C34—C15—C16	99.28 (11)	C36—C35—H35	108.8
N4—C16—C18	115.33 (12)	C34—C35—H35	108.8
N4—C16—C17	114.34 (11)	C37—C36—C35	102.07 (14)
C18—C16—C17	101.24 (11)	C37—C36—H36A	111.4
N4—C16—C15	101.90 (11)	C35—C36—H36A	111.4
C18—C16—C15	115.48 (11)	C37—C36—H36B	111.4
C17—C16—C15	108.92 (11)	C35—C36—H36B	111.4
O1—C17—N3	125.59 (13)	H36A—C36—H36B	109.2
O1—C17—C16	126.48 (13)	C38—C37—C36	104.06 (14)
N3—C17—C16	107.90 (11)	C38—C37—H37A	110.9
C19—C18—C23	119.19 (14)	C36—C37—H37A	110.9
C19—C18—C16	131.86 (13)	C38—C37—H37B	110.9
C23—C18—C16	108.92 (12)	C36—C37—H37B	110.9
C18—C19—C20	118.91 (15)	H37A—C37—H37B	109.0
C18—C19—H19	120.5	N4—C38—C37	104.17 (14)
C20—C19—H19	120.5	N4—C38—H38A	110.9
C21—C20—C19	120.68 (15)	C37—C38—H38A	110.9
C21—C20—H20	119.7	N4—C38—H38B	110.9
C19—C20—H20	119.7	C37—C38—H38B	110.9
C20—C21—C22	121.23 (15)	H38A—C38—H38B	108.9
C20—C21—H21	119.4	C7—N1—C1	114.98 (12)
C22—C21—H21	119.4	C8—N2—C6	114.67 (13)
C23—C22—C21	117.45 (15)	C17—N3—C23	111.20 (12)
C23—C22—H22	121.3	C17—N3—C24	124.95 (13)
C21—C22—H22	121.3	C23—N3—C24	123.85 (12)
C22—C23—C18	122.46 (14)	C16—N4—C38	118.33 (12)
C22—C23—N3	127.28 (14)	C16—N4—C35	110.13 (11)
C18—C23—N3	110.17 (12)	C38—N4—C35	109.36 (12)
N3—C24—C25	113.45 (13)	C33—O2—C32	117.59 (15)
N3—C24—H24A	108.9	H4A—O4—H4B	100.3 (17)
			
N1—C1—C2—C3	179.00 (15)	C21—C22—C23—C18	0.6 (2)
C6—C1—C2—C3	−1.5 (2)	C21—C22—C23—N3	−175.82 (14)
C1—C2—C3—C4	0.1 (3)	C19—C18—C23—C22	−2.5 (2)
C2—C3—C4—C5	1.0 (3)	C16—C18—C23—C22	179.33 (13)
C3—C4—C5—C6	−0.5 (3)	C19—C18—C23—N3	174.42 (12)
C4—C5—C6—N2	177.39 (16)	C16—C18—C23—N3	−3.72 (16)
C4—C5—C6—C1	−1.0 (2)	N3—C24—C25—C26	32.7 (2)
N1—C1—C6—N2	3.1 (2)	N3—C24—C25—C30	−147.57 (19)
C2—C1—C6—N2	−176.36 (14)	C30—C25—C26—C27	0.3 (3)
N1—C1—C6—C5	−178.56 (14)	C24—C25—C26—C27	180.0 (2)
C2—C1—C6—C5	2.0 (2)	C25—C26—C27—C28	0.1 (4)
N1—C7—C8—N2	3.8 (2)	C26—C27—C28—C29	−0.4 (5)
C15—C7—C8—N2	−176.70 (13)	C27—C28—C29—C30	0.3 (5)
N1—C7—C8—C9	−174.74 (13)	C26—C25—C30—C29	−0.3 (4)
C15—C7—C8—C9	4.75 (16)	C24—C25—C30—C29	179.9 (2)
N2—C8—C9—C10	−0.4 (3)	C28—C29—C30—C25	0.1 (5)
C7—C8—C9—C10	178.08 (15)	O3—C33—C34—C35	3.5 (2)
N2—C8—C9—C14	−178.92 (14)	O2—C33—C34—C35	−175.81 (13)
C7—C8—C9—C14	−0.44 (16)	O3—C33—C34—C15	−114.54 (19)
C14—C9—C10—C11	0.6 (2)	O2—C33—C34—C15	66.11 (17)
C8—C9—C10—C11	−177.71 (15)	C7—C15—C34—C33	−70.91 (16)
C9—C10—C11—C12	0.4 (2)	C14—C15—C34—C33	43.49 (16)
C10—C11—C12—C13	−1.3 (2)	C16—C15—C34—C33	164.10 (12)
C11—C12—C13—C14	1.0 (2)	C7—C15—C34—C35	165.19 (12)
C12—C13—C14—C9	0.1 (2)	C14—C15—C34—C35	−80.41 (14)
C12—C13—C14—C15	−177.74 (14)	C16—C15—C34—C35	40.20 (13)
C10—C9—C14—C13	−0.9 (2)	C33—C34—C35—N4	−147.05 (12)
C8—C9—C14—C13	177.77 (13)	C15—C34—C35—N4	−22.83 (14)
C10—C9—C14—C15	177.30 (13)	C33—C34—C35—C36	94.96 (17)
C8—C9—C14—C15	−4.04 (16)	C15—C34—C35—C36	−140.83 (14)
N1—C7—C15—C14	172.83 (13)	N4—C35—C36—C37	29.94 (18)
C8—C7—C15—C14	−6.64 (14)	C34—C35—C36—C37	147.83 (16)
N1—C7—C15—C34	−67.00 (18)	C35—C36—C37—C38	−38.7 (2)
C8—C7—C15—C34	113.53 (13)	C36—C37—C38—N4	32.9 (2)
N1—C7—C15—C16	48.77 (19)	C8—C7—N1—C1	−0.5 (2)
C8—C7—C15—C16	−130.70 (12)	C15—C7—N1—C1	−179.95 (13)
C13—C14—C15—C7	−175.60 (14)	C2—C1—N1—C7	176.80 (14)
C9—C14—C15—C7	6.46 (14)	C6—C1—N1—C7	−2.6 (2)
C13—C14—C15—C34	61.14 (19)	C7—C8—N2—C6	−3.3 (2)
C9—C14—C15—C34	−116.80 (13)	C9—C8—N2—C6	175.00 (14)
C13—C14—C15—C16	−50.2 (2)	C5—C6—N2—C8	−178.33 (14)
C9—C14—C15—C16	131.82 (12)	C1—C6—N2—C8	0.0 (2)
C7—C15—C16—N4	−167.83 (11)	O1—C17—N3—C23	−172.47 (13)
C14—C15—C16—N4	75.26 (13)	C16—C17—N3—C23	5.77 (15)
C34—C15—C16—N4	−43.17 (12)	O1—C17—N3—C24	6.7 (2)
C7—C15—C16—C18	66.38 (16)	C16—C17—N3—C24	−175.06 (12)
C14—C15—C16—C18	−50.53 (16)	C22—C23—N3—C17	175.30 (14)
C34—C15—C16—C18	−168.96 (11)	C18—C23—N3—C17	−1.47 (16)
C7—C15—C16—C17	−46.67 (15)	C22—C23—N3—C24	−3.9 (2)
C14—C15—C16—C17	−163.58 (11)	C18—C23—N3—C24	179.36 (13)
C34—C15—C16—C17	77.99 (12)	C25—C24—N3—C17	−115.58 (16)
N4—C16—C17—O1	46.14 (19)	C25—C24—N3—C23	63.48 (19)
C18—C16—C17—O1	170.79 (14)	C18—C16—N4—C38	−76.15 (16)
C15—C16—C17—O1	−67.08 (17)	C17—C16—N4—C38	40.64 (18)
N4—C16—C17—N3	−132.09 (12)	C15—C16—N4—C38	157.97 (13)
C18—C16—C17—N3	−7.43 (14)	C18—C16—N4—C35	157.07 (12)
C15—C16—C17—N3	114.69 (12)	C17—C16—N4—C35	−86.13 (14)
N4—C16—C18—C19	−47.3 (2)	C15—C16—N4—C35	31.19 (14)
C17—C16—C18—C19	−171.24 (15)	C37—C38—N4—C16	−141.24 (15)
C15—C16—C18—C19	71.31 (19)	C37—C38—N4—C35	−14.10 (18)
N4—C16—C18—C23	130.56 (13)	C36—C35—N4—C16	121.53 (14)
C17—C16—C18—C23	6.58 (14)	C34—C35—N4—C16	−5.40 (15)
C15—C16—C18—C23	−110.87 (13)	C36—C35—N4—C38	−10.11 (17)
C23—C18—C19—C20	2.3 (2)	C34—C35—N4—C38	−137.04 (13)
C16—C18—C19—C20	179.94 (14)	O3—C33—O2—C32	−0.2 (3)
C18—C19—C20—C21	−0.2 (2)	C34—C33—O2—C32	179.15 (16)
C19—C20—C21—C22	−1.7 (2)	C31—C32—O2—C33	88.0 (2)
C20—C21—C22—C23	1.6 (2)		

### Hydrogen-bond geometry (Å, º)

**Table:**

*D*—H···*A*	*D*—H	H···*A*	*D*···*A*	*D*—H···*A*
O4—H4*A*···N2	0.99 (2)	2.03 (2)	2.996 (2)	164 (2)
O4—H4*B*···O1^i^	0.99 (2)	2.31 (2)	3.222 (2)	154 (2)
C2—H2···O1^ii^	0.93	2.50	3.393 (2)	162
C24—H24*A*···O4^iii^	0.97	2.56	3.495 (3)	163

Symmetry codes: (i) *x*+1, *y*, *z*; (ii) −*x*, −*y*+1, −*z*+2; (iii) −*x*+1, −*y*+1, −*z*+2.
